# Light‐induced damage to photosystem II at a very low temperature (195 K) depends on singlet oxygen

**DOI:** 10.1111/ppl.13824

**Published:** 2022-11-28

**Authors:** Heta Mattila, Esa Tyystjärvi

**Affiliations:** ^1^ Department of Life Technologies/Molecular Plant Biology University of Turku Turku Finland

## Abstract

Photosynthetic organisms, like evergreen plants, may encounter strong light at low temperatures. Light, despite being the energy source of photosynthesis, irreversibly damages photosystem II (PSII). We illuminated plant thylakoid membranes and intact cyanobacterial cells at −78.5°C and assayed PSII activity with oxygen evolution or chlorophyll fluorescence, after thawing the sample. Both UV radiation and visible light damaged PSII of pumpkin (*Cucurbita maxima*) thylakoids at −78.5°C, but visible‐light‐induced photoinhibition at −78.5°C, unlike at +20°C, proceeded only in the presence of oxygen. A strong magnetic field that would decrease triplet chlorophyll formation by recombination of the primary radical pair slowed down photoinhibition at −78.5°C, suggesting that singlet oxygen produced via recombination of the primary pair is a major contributor to photoinhibition at −78.5°C. However, a magnetic field did not affect singlet oxygen production at +25°C. Thylakoids of winter leaves of an evergreen plant, *Bergenia*, were less susceptible to photoinhibition both at −78.5°C and +20°C, contained high amounts of carotenoids and produced little singlet oxygen (measured at +20°C), compared to thylakoids of summer leaves. In contrast, high carotenoid amount and low singlet oxygen yield did not protect a *Synechocystis* mutant from photoinhibition at −78.5°C. Thylakoids isolated from *Arabidopsis thaliana* grown under high light, which reduces PSII antenna size, were more resistant than control plants against photoinhibition at −78.5°C but not at +20°C, although carotenoid amounts were similar. The results indicate that visible‐light‐induced photoinhibition at −78.5°C depends on singlet oxygen, whereas photoinhibition at +20°C is largely independent of oxygen.

## 1 INTRODUCTION

Light is the energy source of photosynthesis but also irreversibly damages photosystem II (PSII), and PSII activity is recovered only via degradation and resynthesis of the D1 reaction center protein of the damaged PSII (for reviews, see Theis & Schroda, 2016; Tyystjärvi, 2013). The term photoinhibition refers to such irreversible damage of PSII. UV radiation damages PSII with a high yield (Jones & Kok, 1966), most probably via absorption of UV radiation by the Mn ions of the oxygen‐evolving complex (OEC), which leads to a release of at least one Mn ion (Hakala et al., 2005; Renger et al., 1989; Vass et al., 1996). Many species, if exposed to strong visible light or UV radiation, synthetize UV protectants (e.g., Hakala‐Yatkin et al., 2010; Solanki et al., 2019).

The mechanism by which visible light causes PSII photoinhibition is more controversial (for reviews, see Vass, 2012; Tyystjärvi, 2013; Zavafer & Mancilla, 2021). Absorption of visible light by the Mn ions of the OEC (Hakala et al., 2005; Ohnishi et al., 2005) has been proposed to inactivate PSII. Also oxidative damage, caused by either a long‐lived form of the oxidized primary donor of PSII, P_680_^+^ (Chen et al., 1992; Mattila et al., 2022) or by singlet oxygen generated via PSII charge recombination reactions (e.g., Davis et al., 2016; Mattila et al., 2022; Rehman et al., 2013; Treves et al., 2016), has been suggested to contribute to PSII photoinhibition. In photosynthetic organisms, most singlet oxygen is produced in a reaction between the ground‐state molecular oxygen and an excited triplet chlorophyll (Hideg et al., 1994; Rehman et al., 2013). Triplet chlorophyll, in turn, can be produced either via intersystem crossing in light‐harvesting antennae or via recombination reactions in PSII (or PSI) core. Triplet chlorophylls in the antennae are efficiently quenched by carotenoids (Mozzo et al., 2008). Instead, PSII reaction center chlorophylls are not located close enough to a carotenoid for direct triplet quenching, suggesting that PSII core is the main producer of singlet oxygen in photosynthetic organisms (see also Ramel et al., 2012). However, it has also been suggested that chlorophylls that are not functionally connected to a reaction center contribute to triplet formation (Santabarbara et al., 2001, 2003); such chlorophylls would consequently also contribute to singlet oxygen production.

Many photosynthetic organisms, including evergreen plants (see Solanki et al., 2019), experience high light in combination with so low temperatures that PSII electron transfer reactions are limited to primary charge separation, its rapid reversal, and to reduction of Q_A_. Downregulation of photosynthesis and increased dissipation of excess light energy as heat help plants to combat chilling and freezing stresses (for reviews, see Chang et al., 2021; Fernández‐Marín et al., 2020; Preston & Sandve, 2013). Many species induce a slowly relaxing or sustained form of nonphotochemical quenching (NPQ) of excitation energy (Adams & Demmig‐Adams, 1994; Bag et al., 2020; Demmig‐Adams et al., 2015; Grebe et al., 2020; Malnoë et al., 2018; Míguez et al., 2017; Verhoeven et al., 1996). Low temperature also induces protective mechanisms against reactive oxygen species, like accumulation of xanthophylls and lutein or other antioxidants (e.g., B. Liu et al., 2019; Solanki et al., 2019; X. Wang et al., 2009). These findings confirm that evergreen plants protect their leaves against light during periods of coldness.

Enzymatic reactions of PSII repair usually slow down when temperature drops (Greer et al., 1986), and net photoinhibition occurs when the rate of the damaging reaction is faster than the rate of the repair. Many species are able to enhance the repair after acclimation to a low temperature (e.g., Gombos et al., 1994), though fast repair at very low temperatures, let alone at sub‐zero temperatures, may not be feasible. Not only PSII repair but also the rate of the damaging reaction of photoinhibition decreases with temperature (Lazarova et al., 2014; Mattila et al., 2020, 2022; Tyystjärvi et al., 1994; Ueno et al., 2016), but it is not clear if photoinhibition of PSII occurs at temperatures below zero, or in a frozen environment. A decrease in the amount of the D1 protein has been observed during winter in evergreen spruce needles (Ebbert et al., 2005; Ensminger et al., 2004; Míguez et al., 2017; Ottander et al., 1995; Verhoeven et al., 2009) but the decrease might represent downregulation of photosynthesis rather than PSII damage. However, the accumulation of PSII complexes with properties linking them to the repair cycle has been observed during harsh winter conditions, suggesting ongoing photoinhibition (Grebe et al., 2020). During harsh winters, also low *F*_V_/*F*_M_ values are observed, especially when high light coincides with a low temperature (Ebbert et al., 2005; Grebe et al., 2020), but the low *F*_V_/*F*_M_ is, at least mostly, due to formation of strong sustained NPQ, and therefore this fluorescence parameter cannot be directly used to estimate PSII photoinhibition in low‐temperature conditions. It was found, however, that *F*_V_/*F*_M_ did not completely recover after transferring the needles to above‐zero temperatures, suggesting that some PSII centers were actually photoinhibited (Grebe et al., 2020). On the other hand, during mild winters, spruce seems to prevent the damage or repair it, as the *F*_V_/*F*_M_ values have been reported to stay high (Grebe et al., 2020). However, questions remain about whether photoinhibition occurs at very low temperatures, and if so, how fast it proceeds and what its mechanism is.

Here, we measured photoinhibition of PSII at −78.5°C. An ultra‐low temperature was chosen to make sure that PSII charge recombination reactions, except for the rapid recombination of the primary radical pair, are negligibly slow (Rappaport & Lavergne, 2009; Zabelin et al., 2016). Furthermore, singlet oxygen is expected to be the main reactive oxygen species formed at an ultra‐low temperature because chemical reactions required for one‐electron reduction of oxygen are expected to be slow. Photoinhibition was mostly assayed, after melting the sample, by measuring the light‐saturated oxygen evolving capacity of PSII in the presence of an artificial electron acceptor to exclude the possibility that sustained quenching, possibly induced by the treatment, would have affected the results. Using thylakoids also excludes interferences from the ongoing repair reactions. To obtain insights into the mechanism, experiments were conducted both in the presence and absence of oxygen. In addition, the effects of antenna size, carotenoid amount and singlet oxygen production rate were tested. Basic data were obtained with thylakoids isolated from pumpkin but *Arabidopsis thaliana* thylakoids and cells of the cyanobacterium *Synechocystis* sp. PCC 6803 were used to assess the effects of antenna size and carotenoids on low temperature photoinhibition. Thylakoids were also isolated from winter leaves of the evergreen plant *Bergenia* to see if plants that stay green during winter can tolerate photoinhibition at −78.5°C.

## 2 MATERIALS AND METHODS

### 2.1 Organisms and growth conditions

Pumpkin (*Cucurbita maxima* L.), *A. thaliana* (Columbia‐0) and *Synechocystis* sp. PCC 6803 were grown in growth chambers (Weiss Gallenkamp) at 20°C (the plants) or at 32°C (*Synechocystis*), in a 16‐h (pumpkin) or 8‐h (*Arabidopsis*) light period or under constant light (*Synechocystis*) of photosynthetic photon flux density (PPFD) 150–200 μmol m^−2^ s^−1^ (the plants) or 40 μmol m^−2^ s^−1^ (*Synechocystis*) from fluorescent lamps. Some of the *Arabidopsis* plants having the first pair of real leaves were transferred to high light conditions (PPFD 1000 μmol m^−2^ s^−1^) for 10 weeks, as described in Mattila et al. (2020). *Synechocystis* cells were grown in BG‐11 liquid medium (Rippka et al., 1979), supplied with 20 mM Hepes‐NaOH (pH 7.5). The ΔrpoZ mutant strain is described in Gunnelius et al. (2014).

Leaves from outdoors‐grown *Bergenia* were collected on March 1, 2018, June 11, 2018, March 10, 2019, June 5, 2019, February 18, 2021, July 7, 2021, August 18, 2021, and December 10, 2021 (Nousiainen, Finland). In some figures, the Roman numbers I and II are used to signify spring (February 18) and autumn (December 10), respectively, for the year 2021. An overview of the weather during 2 weeks preceding the winter collection days is shown in Table 1.

**TABLE 1:** Averages of air temperature, maximum daily air temperature, thickness of the snow cover, and an estimate of the cloud coverage (where 1 refers to no clouds and 8 to maximum cover) over one and two (in parenthesis) weeks preceding the collection of leaves on the indicated days. The weather data were measured by the Finnish meteorological institute in Artukainen (Turku, Finland, 24.5 km from the collection site in Nousiainen). According to personal observations during several years, the temperature at the collection site in Nousiainen is typically 1–2°C lower than in Turku.

Collection date	Weather parameters			
	Average temperature (°C)	Average daily maximum (°C)	Snow cover (cm)	Cloudiness, rel. Between 1–8
Mar 1, 2018	−12.7 (−10.1)	−9.0 (−6.7)	13.8 (13.9)	2 (3)
Mar 10, 2019	−2.5 (−0.5)	−0.3 (1.7)	12.4 (13.0)	6 (5)
Feb 18, 2021	−9.3 (−10.2)	−5.0 (−6.8)	25.3 (25.1)	4 (3)
Dec 10, 2021	−11.4 (−9.3)	−10.7 (−8.6)	2.8 (2.5)	4 (4)

### 2.2 Thylakoid isolation

Thylakoid membranes were isolated from leaves as described in Hakala et al. (2005), with modifications described in Mattila et al. (2020), and stored at −75°C. Prior to the isolation, pumpkin plants were kept in the dark for 24 h to lower the amount of starch in leaves. *Bergenia* leaves, when collected during winter, were kept for 13 (2018) or for 3–4 days (2019 and 2021) at −20°C in the dark, or, when collected during summer, for few hours at room temperature in the dark. *Arabidopsis* thylakoids were isolated directly from growth conditions. *Bergenia* thylakoids isolated on December 10, 2021 were not homogenous and those thylakoids were additionally filtered through Miracloth (Merck Millipore) before experiments. A batch of thylakoids was isolated from at least three leaves (*Bergenia*) or from leaves belonging to at least three plant individuals (pumpkin and *Arabidopsis*).

### 2.3 Pigment analyses

Chlorophylls *a* and *b* and carotenoids were extracted from isolated thylakoids in 80% acetone (buffered to pH 7.8) and from leaf discs (*d* = 6 mm) in dimethylformamide. After addition of the solvent, thylakoid samples were centrifuged (10 min, 10,000 *g*) and immediately analyzed. Leaf samples were kept at 4°C in the dark for 4–20 days, until the leaf discs became completely white, after which the samples were analyzed. Chlorophylls were quantified spectrophotometrically according to Porra et al. (1989) and carotenoids according to Wellburn (1994).

### 2.4 Photoinhibition treatments

**FIGURE 1:**
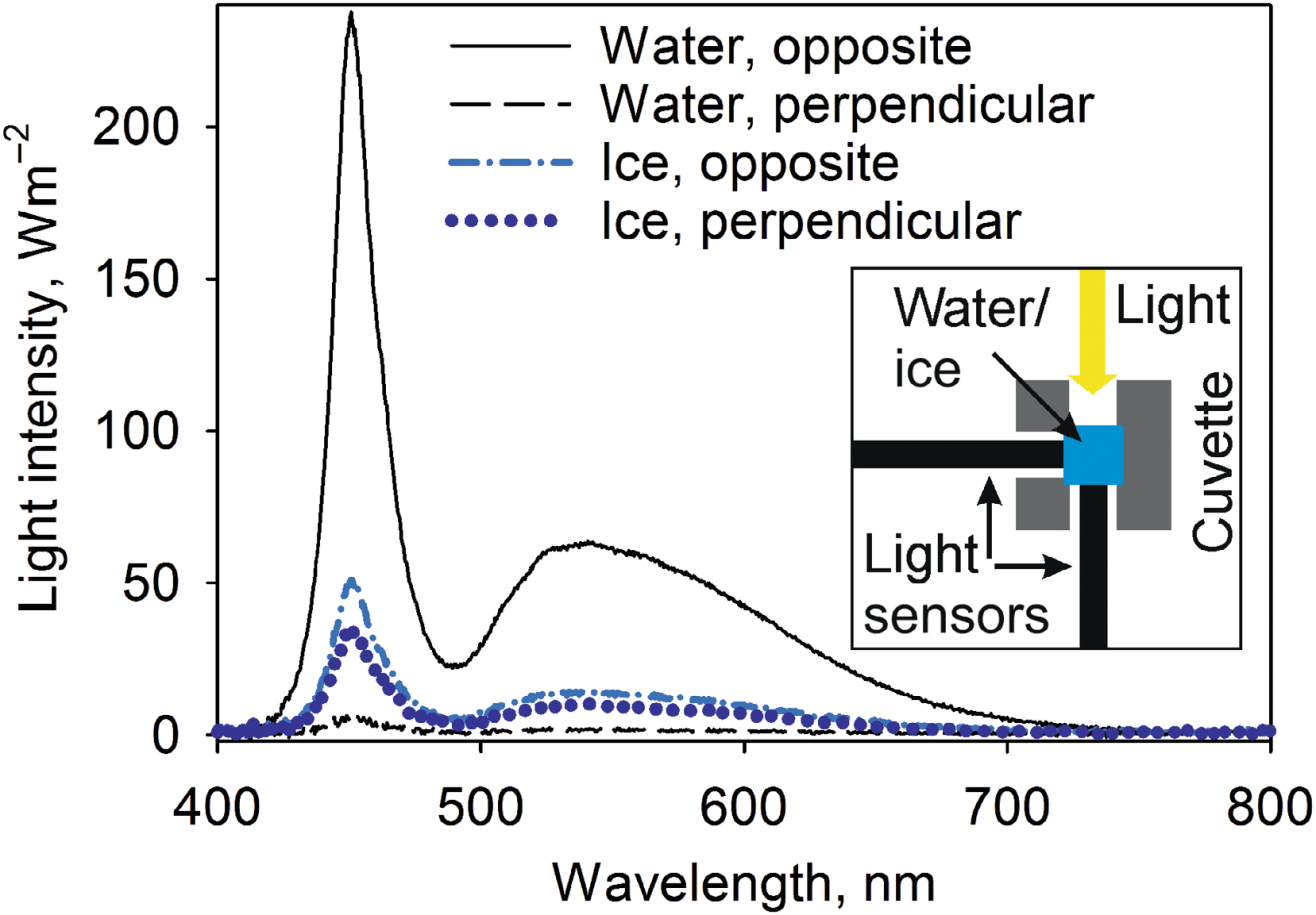
Transmission of light through water or ice. A 1‐ml cuvette, filled either with water (black lines) or ice (blue lines), was illuminated from a side with a cold white LED, and the spectra were measured both from the opposite and the perpendicular sides of the cuvette with a spectrophotometer, as drawn in the inset (the blue square represents a cuvette filled with water or ice, the yellow arrow a light source, the gray areas an opaque cuvette holder and the black bars the two positions of the spectrophotometer light sensor; a top view).

Thylakoids (100 μg chlorophylls [*a* + *b*] ml^−1^) were illuminated in a photoinhibition buffer (40 mM HEPES‐KOH [pH 7.4], 1 M betaine monohydrate, 330 mM sorbitol, 5 mM MgCl_2_, and 5 mM NaCl), and intact *Synechocystis* cells (optical density at 730 nm [OD730] = 0.44–0.48) in BG‐11 with 10% glycerol, for 40–240 min, either at −78.5°C or +20°C, as indicated in the respective figure legends. White light was obtained with a cold white LED (PPFD 1000–2000 μmol m^−2^ s^−1^, as indicated; for the spectrum, see Figure 1) and UV‐A radiation (365 nm; PFD 300 μmol m^−2^ s^−1^) with VL‐8.LC lamp (Vilber Lourmat). Before illumination at −78.5°C, the sample (0.4–1 ml) was prepared in a spectrophotometer cuvette and frozen inside a −75°C freezer. Illumination treatments were performed while surrounding the cuvette with pellets of dry ice. The effect of the white light illumination on the temperature of the sample was tested; the temperature stayed below −60°C. When the treatment was done at 20°C (liquid sample), the sample was continuously mixed, either with a magnetic stirrer or by constantly flushing the sample with air. Anaerobicity was achieved by first flushing the sample with nitrogen gas for 3 min and then adding 6 mM glucose, 8 U ml^−1^ glucose oxidase and 800 U ml^−1^ catalase, after which the sample was, in the case of photoinhibition at −78.5°C, frozen, and in the case of photoinhibition at 20°C, directly treated.

External magnetic field (~170 mT or 1700 G) was achieved by placing a cuvette between two strong permanent magnets (BY0Y0X0‐N52, size 2 × 2 × 1 in., K&J Magnetics, Inc.; see Hakala‐Yatkin et al., 2011), placed 1 cm apart from each other. In the control experiments, the sample was placed between two iron blocks of similar sizes.

### 2.5 Quantification of photoinhibition

PSII activity was measured, before and after an illumination treatment or after a control treatment in the dark by recording the rate of light‐saturated (i.e., maximum) oxygen evolution capacity of PSII in the presence of artificial electron acceptors (0.5 mM 2,6‐dimethylbenzoquinone [DMBQ], additionally with 0.5 mM hexacyanoferrate[III] with *Synechocystis*) from aliquots of the treated sample. The oxygen evolution measurement was conducted either at 22°C (plant thylakoids) or at 32°C (*Synechocystis*) with an oxygen electrode (Hansatech). When glucose oxidase was used for the removal of oxygen, oxygen consumption by glucose oxidase was first quantified in the dark and the value was added to the oxygen evolution of PSII measured subsequently in the light.

In some cases, as indicated, PSII activity was also estimated by measuring the fluorescence parameter (*F*_M_ − *F*_0_)/*F*_M_ (=*F*_V_/*F*_M_). In the case of *Synechocystis* cells, *F*_V_/*F*_M_ values were measured after 20 min in the dark at room temperature with Aquapen (Photon Systems Instruments). *F*_V_/*F*_M_ values from leaves (at least 30 min in the dark) or from plant thylakoids (5 min in the dark) were measured with FluorPen (Photon Systems Instruments).

In the case of experiments conducted at 20°C, the rate constant of photoinhibition (*k*_PI_) was calculated in Excel (Microsoft) by assuming that the loss of oxygen evolution capacity of PSII or the decrease in *F*_V_/*F*_M_ follows first‐order reaction kinetics (see Tyystjärvi, 2013). In the case of aerobic photoinhibition at 20°C, the loss of oxygen evolution capacity of PSII after several time‐points of the treatment was fitted to the first‐order reaction equation with SigmaPlot (Systat Software Inc.). The rate constant of PSII inactivation in the dark (*k*_DARK_) was always measured and subtracted from raw rate constants to obtain the final *k*_PI_ value.

The kinetic pattern of photoinhibition at −78.5°C was determined by using pumpkin thylakoids. Inactivation of PSII by freezing and thawing in the dark was taken into account by using the oxygen evolution activity of a frozen‐and‐thawed sample as the control value. To obtain the dependence of the rate constant (initial rate) of the reaction on the observed loss of PSII activity, we built a model in which a finite initial amount of oxygen is being converted, in the light, to singlet oxygen which, in turn, can be converted back to O_2_, cause loss of PSII activity, or cause unspecified oxidations. Photoinhibition and unspecified oxidations together lead to depletion of O_2_ (Figure S1). Conversion of O_2_ to singlet oxygen and back conversion, as well as the loss of singlet oxygen due to unspecific oxidations, were treated as first‐order reactions and photoinhibition as a second‐order reaction (PSII + singlet oxygen → inactive PSII). The rate constants of the four reactions were let to run free when modeling the kinetics, but when the model was later used for the calculation of a relative value for the rate constant of photoinhibition at −78.5°C from fixed‐time assays, all other rate constants except that for the conversion of O_2_ to singlet oxygen were fixed to the values obtained from the kinetic experiment, thus assuming that the rate of photoinhibition is directly proportional to the rate of singlet oxygen generation when samples of the same type are compared. Version 4.37 of the Copasi software (Hoops et al., 2006) was used for the modeling.

### 2.6 Western blot

After illumination, 0.2 mM guanosine‐5'‐triphosphate (GTP) was added to the thawed thylakoid sample to enhance degradation of the D1 protein after photo‐inactivation. Samples of 20 μl (0.05 μg chlorophyll ml^−1^, diluted with the photoinhibition buffer and sample loading buffer [4×] from NEXT GEL®) were loaded and proteins separated with the 10% NEXT GEL® SDS‐PAGE (Amresco), as described by Hakkila et al. (2013). The D1 protein was detected with the AS06124A antibody (1:10,000 dilution; Agrisera) and CDP Star Chemiluminescence Kit (New England Biolabs) as described in Hakkila et al. (2013).

### 2.7 Singlet oxygen measurements

Singlet oxygen was measured with the method initially used by Telfer et al. (1994) with the modifications described in Rehman et al. (2013). Isolated thylakoid membranes (80 μg chlorophyll ml^−1^ with *Bergenia*; 100 μg chlorophyll ml^−1^ with pumpkin) were illuminated (PPFD 3000 μmol m^−2^ s^−1^ of white light from a halogen lamp for *Bergenia* or from a sunlight simulator (SL Holland) for pumpkin), in the absence or presence of 20 mM L‐histidine at 22 or 25°C in the photoinhibition buffer. Oxygen concentration was recorded by an oxygen electrode (Hansatech; *Bergenia*) or with an optode (Firesting, Pyro Science; pumpkin), as described in Mattila et al. (2022). Oxygen consumption in the absence of added histidine was subtracted from the oxygen consumption in the presence of histidine to obtain the final values. Additionally, singlet oxygen was detected with Singlet Oxygen Sensor Green (SOSG; Invitrogen™). Thylakoids (100 μg chlorophyll ml^−1^) were illuminated by red (PPFD 1000 μmol m^−2^ s^−1^; *λ* > 650 nm obtained with the long‐pass edge filter LL‐650; Corion) light for 15 min at 25°C in the presence of 2 μM SOSG. Before and after the illumination, SOSG fluorescence (excited with 500 nm light, obtained with a Corion band‐pass filter with 10 nm full width at half maximum) at 530 nm was recorded with a QE Pro spectrometer (Ocean Insight).

### 2.8 Statistical tests

All experiments with biological material were repeated at least three times. In the case of pumpkin and *Bergenia* thylakoids, repetitions originated from a thylakoid batch and in the case of *Arabidopsis*, from three independently isolated thylakoid batches. In the case of leaves, a repetition always represents an individual leaf. Student *t* tests were calculated in Microsoft Excel (two‐tailed, heteroscedastic). A linear model for the loss of the D1 protein was constructed in R version 4.13 (R Core Team, 2021), using the lme4 library (Bates et al., 2015). See Appendix S1 for details.

## 3 RESULTS

### 3.1 PSII photoinhibition at −78.5°C depends on oxygen

To test if photoinhibition occurs at low temperatures, thylakoid membranes, isolated from leaves of greenhouse‐grown pumpkin, were first frozen (at −75°C) and then illuminated with high light while buried in dry ice (−78.5°C or 195 K). PSII activity was quantified, both before freezing the sample and after the illumination treatment, by measuring the light‐saturated rate of oxygen evolution of PSII (H_2_O to DMBQ) at 22°C. PSII activity was also measured from samples that were frozen (at −75°C) and thawed in the dark to quantify the amount of dark‐inactivation to PSII caused mainly by freezing and thawing.

**FIGURE 2:**
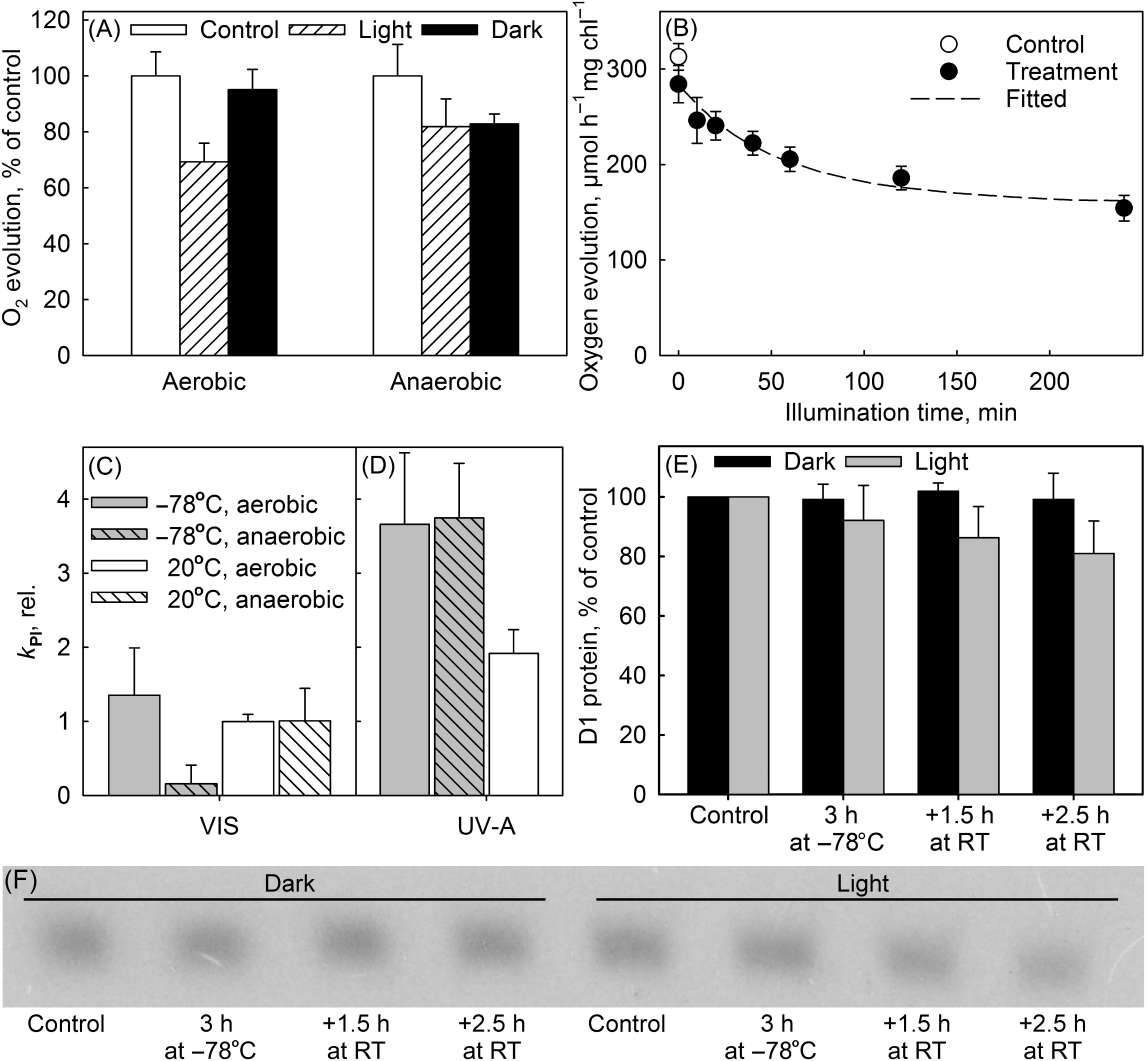
Photoinhibition of photosystem II (PSII) in isolated thylakoid membranes of pumpkin. (A) Thylakoid membranes were illuminated with white light (photosynthetic photon flux density [PPFD] 1150 μmol m^−2^ s^−1^) for 40 min at −78.5°C in aerobic or anaerobic conditions, as indicated, after which PSII activity was assayed with oxygen evolution (H_2_O to dimethylbenzoquinone). The dark sample has been frozen/thawed in the dark. (B) Thylakoid membranes were illuminated with white light (PPFD 1150 μmol m^−2^ s^−1^) for 0–240 min at −78.5°C (solid symbols) in aerobic conditions. The first sample (0 min) has been frozen/thawed in the dark. The open symbol (control) shows PSII activity before any treatment. The dashed line shows the best fit to a model described in Figure S1. The experiment in (A) was done with a different batch of thylakoids and is not a part of the kinetic experiment. (C) Thylakoid membranes were illuminated with white light (VIS) for 40 min (PPFD 1150 μmol m^−2^ s^−1^), either at −78.5°C (gray bars) or at 20°C (white bars), in aerobic (open bars) or anaerobic (hatched bars) conditions. Rate constants of photoinhibition (*k*_PI_) were calculated assuming that a decline in the PSII oxygen evolution rate followed the kinetics shown in (B). The rate constants of photoinhibition at −78.5°C are normalized to that obtained by modeling the result from (B), and the rate constants of photoinhibition at 20°C to that obtained under aerobic conditions at 20°C, to facilitate comparison. For original PSII activities, see Figure S2. (D) Thylakoid membranes were illuminated with UV‐A radiation for 10 min (PFD 300 μmol m^−2^ s^−1^) in aerobic or anaerobic conditions. (E) Quantification of the D1 protein from western blots of untreated thylakoid samples (Control) and from samples incubated in the dark (black bars) or illuminated with white light (PPFD 1150 μmol m^−2^ s^−1^; gray bars) for 3 h, at −78.5°C. After the treatments, samples were melted and incubated at room temperature (RT) for 1.5 or 2.5 h, as indicated. The data show averages from at least three independent repetitions and the error bars show SD. (F) A representative western blot.

Clear damage to PSII was observed after 40‐min illumination with white light at −78.5°C (Figure 2A). However, when oxygen was removed with a glucose oxidase treatment prior to the illumination or dark incubation, almost no photoinhibition was observed at −78.5°C after white light illumination. The difference between aerobic and anaerobic illumination was statistically significant (*p* = 0.005; Student *t* test). The dependence of photoinhibition at −78.5°C on oxygen suggests the involvement of reactive oxygen species, most probably singlet oxygen. Photoinhibition at −78.5°C clearly deviated from first‐order reaction kinetics as the rate of photoinhibition was fast in the beginning but slowed down with time, eventually approaching zero (Figure 2B). The stabilization of PSII activity during illumination suggests that oxygen in the vicinity of PSII is depleted during the illumination of the frozen sample. A model of the kinetics of photoinhibition at −78.5°C was built by assuming that a finite amount of oxygen is available for a reaction that causes photoinhibition of PSII via conversion of oxygen to singlet oxygen. Depletion of oxygen was assumed to also involve unspecified reactions (not leading to PSII inactivation) of singlet oxygen (see Figure S1 for the details of the model). The model successfully reproduced the kinetics of photoinhibition at −78.5°C (Figure 2B). For the analysis of the fixed‐time assays, all rate constants of the model, except that for singlet oxygen production, were fixed to the values obtained from the original fitting of the kinetics.

In addition, thylakoids were illuminated at 20°C with constant mixing during the illumination or dark incubation. In this case, the decrease in PSII oxygen evolution capacity was fitted to a first‐order reaction equation to calculate rate constants. The rate constant of dark inactivation was subtracted from the rate constant obtained from an illuminated sample to obtain the rate constant of photoinhibition (*k*_PI_). Oxygen removal had no effect on photoinhibition at 20°C (Figure 2C); the behavior was in sharp contrast to photoinhibition at −78.5°C. Illumination with UV‐A radiation caused photoinhibition both at −78.5°C and 20°C, with a higher yield than visible light, and anaerobicity did not affect photoinhibition induced by UV‐A at −78.5°C (Figure 2B).

UV‐A caused photoinhibition with a higher yield than visible light both at −78.5°C and 20°C, suggesting that a similar mechanism functions at both temperatures, but when photoinhibition was induced with visible light, oxygen removal had contrasting effects on photoinhibition at these temperatures (Figure 2C,D). To test whether the decrease in PSII activity at −78.5°C has similar molecular consequences as photoinhibition at above‐zero temperatures, we measured the amount of the D1 protein after photoinhibition at −78.5°C with western blotting. The amount of the D1 protein was also measured after freezing and thawing the sample in the dark to ensure that the observed effects were caused by the low‐temperature illumination. GTP was added to thylakoids after illumination as it is needed for the enzymatic degradation of the D1 protein (Spetea et al., 1999). Indeed, slow degradation of the D1 protein was observed after illumination at −78.5°C and subsequent incubation of the thylakoid sample at room temperature. Stable levels of the D1 protein in nonilluminated control samples confirmed that degradation of the D1 protein was triggered by illumination also at −78.5°C (Figure 2E,F). A linear statistical model of the relative amount of the D1 protein, with the illumination time, dark time and their interaction as explanatory variables, was highly significant (*F*(3,26) = 9.883, *p* = 0.00016; see Appendix S1 for the full characterization of the model) and showed that the lowering of the amount of the D1 protein due to interaction by illumination and subsequent dark time was significant (*p* = 0.0485); the negative effect of illumination alone on the D1 protein was not significant (*p* = 0.0791), and dark time alone had no effect (*p* = 0.997).

Besides the different kinetics (Figure 2B), a direct comparison between the rates of photoinhibition at −78.5 and 20°C is complicated by the fact that ice strongly scatters light (Figure 1), and therefore thylakoids within frozen samples at −78.5°C are expected to receive less light than similar liquid suspensions.

### 3.2 Magnetic field protects against photoinhibition at −78.5°C

Photoinhibition kinetics and dependence on oxygen suggest that a reactive oxygen species, most probably singlet oxygen, is decisive for photoinhibition at −78.5°C but does not tell whether singlet oxygen is produced by recombination reactions or by intersystem crossing in the light‐harvesting antenna of PSII. At −78.5°C, the only recombination reaction that might produce triplet chlorophyll with a significant rate is the recombination of the primary charge pair (P_680_^+^Pheo^−^) right after charge separation. We probed the importance of this recombination reaction by illuminating samples in a strong external magnetic field. A magnetic field is expected to lower the triplet yield of the recombination by fixing the axis of spin precession. This lowers the triplet yield because only the middle triplet energy level becomes populated (Hoff et al., 1977). The expected magnetic field‐induced lowering of singlet oxygen production has been confirmed during the illumination of quinone‐depleted reaction centers of a carotenoid‐less mutant of the photosynthetic bacterium *Rhodobacter sphaeroides* (Y. Liu et al., 2005).

**FIGURE 3:**
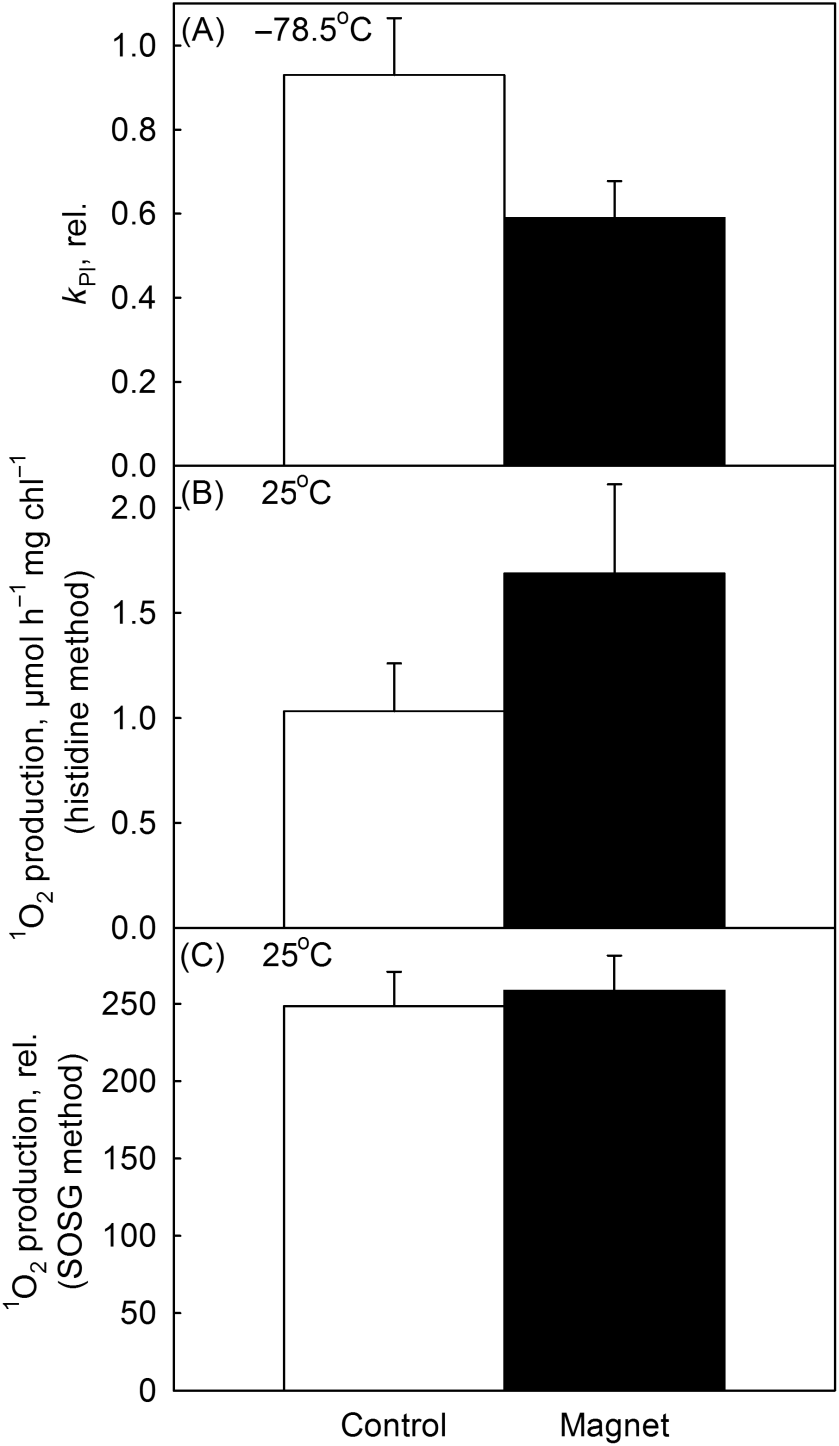
Photoinhibition and singlet oxygen production in the absence (Control) and presence (Magnet) of a 170 mT external magnetic field in pumpkin thylakoids. (A) Thylakoids were illuminated with white light (PPFD 1150 μmol m^−2^ s^−1^) at −78.5°C for 40 min. Rate constants of photoinhibition (*k*_PI_) were calculated assuming that a decline in the PSII oxygen evolution rate (H_2_O to dimethylbenzoquinone) followed the kinetics shown in Figure 2B. The rate constants are normalized to that obtained by modeling the result from Figure 2B. For original PSII activities, see Figure S3. (B) Singlet oxygen production in high white light (PPFD 3000 μmol m^−2^ s^−1^) at 25°C, measured with a histidine‐based method. (C) Singlet oxygen production in strong red light (PPFD 1000 μmol m^−2^ s^−1^; *λ* > 650 nm) at 25°C, measured with SOSG. Each bar represents an average of at least three independent repetitions and the error bars show SD.

Illumination of pumpkin thylakoids at −78.5°C in the presence and absence of a strong magnetic field revealed that the rate constant of photoinhibition in a magnetic field was only 63.5% of the rate constant measured in the absence of a strong field (Figure 3A). According to Student *t* test, the result was statistically significant (*p* = 0.03). This result strongly suggests that photoinhibition at −78.5°C depends on singlet oxygen produced via the recombination of the primary pair.

We also measured singlet oxygen production at 25°C with the histidine method and with a fluorescent probe (SOSG). Both methods showed that an external magnetic field did not lower singlet oxygen production (Figure 3B,C), seemingly contrasting with the photoinhibition results observed at −78.5°C. The simplest explanation for the lack of a magnetic field effect at 25°C is that in PSII (contrary to reaction centers of nonoxygenic photosynthetic bacteria), the recombination of the primary charge pair after charge separation is not the main producer of singlet oxygen at physiological temperatures.

### 3.3 Winter thylakoids of *Bergenia* are tolerant against photoinhibition at −78.5°C

While −78.5°C is an extreme temperature, many photosynthetic organisms do experience low temperatures in combination with high light. One such example is *Bergenia*, a flowering evergreen dicot native to central Asia and a popular garden plant in Finland. We collected green *Bergenia* leaves during several summers and winters for isolation of thylakoid membranes. During winter, leaves were collected after periods of cold weather; the maximum (warmest) day‐time air temperature during the two‐week period preceding the collection varied from −0.3 to −14.7°C (2018, spring), from 0 to −11.6°C (2021, spring), and from −1.4 to −16°C (2021, autumn).

**FIGURE 4:**
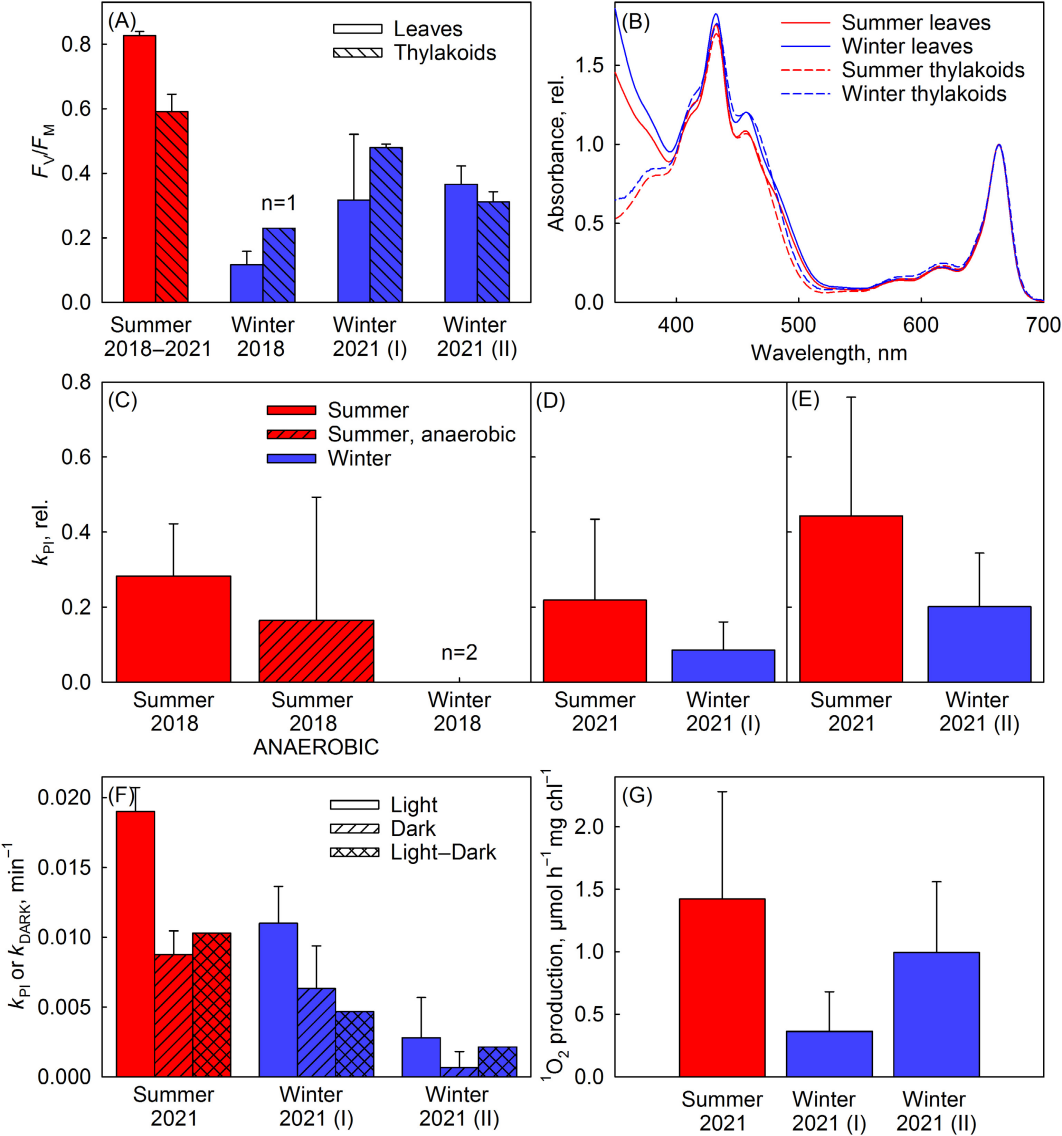
Photoinhibition in *Bergenia* thylakoids, isolated from leaves collected either during winter or summer. (A) *F*_V_/*F*_M_ values were measured from leaves (open bars) and thylakoids (hatched bars) after at least 30 min (leaves) or 5 min (thylakoids) in the dark, collected on summer (red bars) or winter (blue bars) on the indicated years. (B) Absorption spectra, normalized to 665 nm, of leaf (in dimethylformamide; continuous lines) and thylakoid (in acetone; dashed lines) extracts. Leaves were collected in summer (red lines) or winter (blue lines) 2021. The chlorophyll contents of the leaves were 38.0 ± 3.25 μg chl cm^−2^ (summer) and 42.7 ± 5.4 μg chl cm^−2^ (winter). (C) Summer and winter thylakoids were illuminated at −78.5°C (photosynthetic photon flux density [PPFD] 2000 μmol m^−2^ s^−1^) for 120 min. If not otherwise indicated with the label “ANAEROBIC,” illumination was conducted in the presence of oxygen. Rate constants of photoinhibition (*k*_PI_) were calculated assuming that a decline in the PSII oxygen evolution rate (H_2_O to dimethylbenzoquinone) followed the kinetics shown in Figure 2B. The rate constants are normalized to that obtained by modeling the result from Figure 2B. For original PSII activities, see Figure S4. (D, E) Repetition of photoinhibition at −78.5°C with different thylakoid batches. (F) Thylakoids were illuminated (PPFD 2000 μmol m^−2^ s^−1^; Light) or incubated in the dark (Dark) at 20°C for 45 min. Rate constants were calculated assuming that a decline in the PSII oxygen evolution rate followed, both in the light and in darkness, first‐order reaction kinetics; rate constants of photoinhibition (*k*_PI_; Light–Dark) were obtained by subtracting the rate of dark‐inactivation (*k*_DARK_) from the rate of light induced decline. (G) Singlet oxygen production in high light (PPFD 3000 μmol m^−2^ s^−1^) at 22°C by thylakoid membranes, measured with a histidine‐based method. The Roman numbers I and II after 2021 refer to spring and autumn, respectively. All data show averages from at least three independent repetitions (unless otherwise specified) and error bars show SD.

The PSII activity of winter leaves, estimated by the fluorescence parameter *F*_V_/*F*_M_, measured after at least 30 min of darkness, was 15%–44% of that of summer leaves (Figure 4A), and the PSII activity of thylakoids (*F*_V_/*F*_M_ was measured after at least 5 min of darkness), isolated from winter leaves, was 39%–81% of that of thylakoids isolated from summer leaves (Figure 4A). We did not test if the *F*_V_/*F*_M_ values measured from winter leaves would recover if leaves would be kept at warmer conditions, but the finding that the *F*_V_/*F*_M_ values measured from winter thylakoids were in most cases higher than those of winter leaves, suggests that the winter leaves of *Bergenia* may have had sustained quenching of excitation energy that was not fully preserved in thylakoid isolation. Winter leaves (*p* = 1.1× 10^−10^ according to a *t* test) and thylakoid membranes (*p* = 6.6 × 10^−9^) isolated from winter leaves contained more carotenoids and unidentified UV‐absorbing compounds than summer leaves (Figure 4B).

Next, we illuminated *Bergenia* thylakoids, isolated from both winter and summer leaves, with white light at −78.5°C. A clear decrease in PSII activity, measured as oxygen evolution, was observed in summer thylakoids, but the *k*_PI_ values were smaller than those of pumpkin thylakoids (Figures 2 and 4D,E). Anaerobicity seemed to lower the rate constant of photoinhibition at −78.5°C, similarly as in pumpkin thylakoids (Figures 2 and 4C), though the difference was not statistically significant in *Bergenia* thylakoids. Interestingly, thylakoids of winter‐harvested *Bergenia* were even more tolerant against photoinhibition at −78.5°C than thylakoids of summer‐harvested leaves (*p* = 0.03); no photoinhibition was observed in thylakoids isolated during winter 2018 (but only two experiments could be done due to a small amount of plant material), and the *k*_PI_ values of thylakoids isolated during winter 2021 were ~50% of those of summer thylakoids (Figure 4D,E).

Thylakoids were illuminated also at 20°C, and we observed that, in addition to being tolerant against photoinhibition at −78.5°C, winter thylakoids were more tolerant against photoinhibition at 20°C than summer thylakoids (*p* = 0.005), especially thylakoids isolated during winter/autumn 2021 (Figure 4F). Last, singlet oxygen production in high light at 20°C was measured from the thylakoids with a histidine‐based method. Thylakoids isolated from winter leaves appeared to produce less singlet oxygen than summer thylakoids (Figure 4G) but the differences were not statistically significant.

Many properties of *Bergenia* winter leaves and thylakoids varied between the collection years (Figure 4). The result is expected as temperature and light conditions vary between years (see Table 1). Specifically, it has been shown that evergreen plants are affected by the thickness of snow cover and the melting time of snow during spring (Lundell et al., 2010; Solanki et al., 2019).

### 3.4 High carotenoid amount of a cyanobacterial mutant did not protect it from photoinhibition at −78.5°C

Were *Bergenia* thylakoids isolated from winter leaves (partly) protected against photoinhibition at −78.5°C because they contained high amounts of carotenoids and produced little singlet oxygen at 20°C? The idea was further studied with the help of the ΔrpoZ strain of the cyanobacterium *Synechocystis* sp. PCC 6803 (Gunnelius et al., 2014). The strain lacks the ω subunit of the RNA polymerase, grows normally under optimal conditions and possesses PSII, PSI and the phycobilisome antenna in similar stoichiometry as the control strain but contains twice as much β‐carotene as the control strain and also elevated amounts of other carotenoids and α‐tocopherol (Gunnelius et al., 2014). In agreement with the high carotenoid levels, the ΔrpoZ strain produces less singlet oxygen (Kurkela et al., 2017) and exhibits lower rates of photoinhibition (Hakkila et al., 2013) at 32°C than the control strain.

**FIGURE 5:**
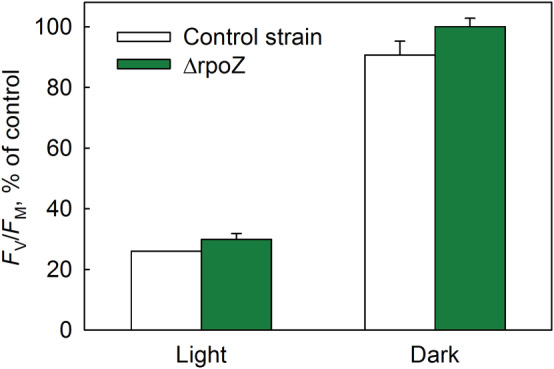
Effect of carotenoids on photoinhibition at −78.5°C. Intact cells of the control and the mutant strain (ΔrpoZ) of *Synechocystis* were illuminated (photosynthetic photon flux density 1000 μmol m^−2^ s^−1^; Light) for 60 min or frozen/thawed in the dark (Dark) in the presence of 10% glycerol. Photosystem II activity was assayed with the *F*_V_/*F*_M_ parameter, measured after 20 min in the dark at room temperature. Control *F*_V_/*F*_M_ values, before treatments, were 0.5 ± 0 and 0.36 ± 0.006 for the control and the ΔrpoZ strain, respectively. Each bar represents an average of at least three independent repetitions and the error bars show SD.

The ΔrpoZ mutant and the control strain of *Synechocystis* were frozen (in 10% glycerol to protect against unspecific damage during freezing and thawing) and illuminated at −78.5°C. As earlier observed (Gunnelius et al., 2014), the ΔrpoZ strain showed a lower PSII activity, compared to the control strain, before any treatments; light‐saturated PSII oxygen evolution rates were 2.6 ± 0.48 and 2.0 ± 0.21 μmol O_2_ h^−1^ OD730^−1^ for the control and the ΔrpoZ strains, respectively. After freezing (at −75°C) and thawing in the dark, the PSII oxygen evolution rates of the control strain decreased to 20% of the original values, whereas in the ΔrpoZ strain the PSII oxygen evolution rates decreased only to 66%. The ΔrpoZ strain contains high amounts of glycogen (Kurkela et al., 2017), which may have protected the OEC against freezing and melting. Previously, it has been shown that the OEC of PSII is especially vulnerable to freezing/thawing (W.Q. Wang et al., 1992). As the PSII oxygen evolution capacity decreased dramatically already without any illumination in the control strain, photoinhibition at −78.5°C was quantified with the fluorescence method. *F*_V_/*F*_M_ values declined due to freezing and thawing in the dark only a little in the control strain, and not at all in the ΔrpoZ strain (Figure 5). In contrast to photoinhibition at 32°C (Hakkila et al., 2013), illumination at −78.5°C inactivated PSII at a similar rate in both strains (Figure 5), showing that the high amount of carotenoids and slow singlet oxygen production at 32°C did not offer protection at −78.5°C. As the total extent of photoinhibition at −78.5°C was larger in *Synechocystis* than in plant thylakoids (Figures S2, S3, and 5), no reliable calculations of the rate constant could be done with our model.

### 3.5 Thylakoids of high light‐grown *Arabidopsis* were tolerant against photoinhibition at −78.5°C but not at 20°C

**FIGURE 6:**
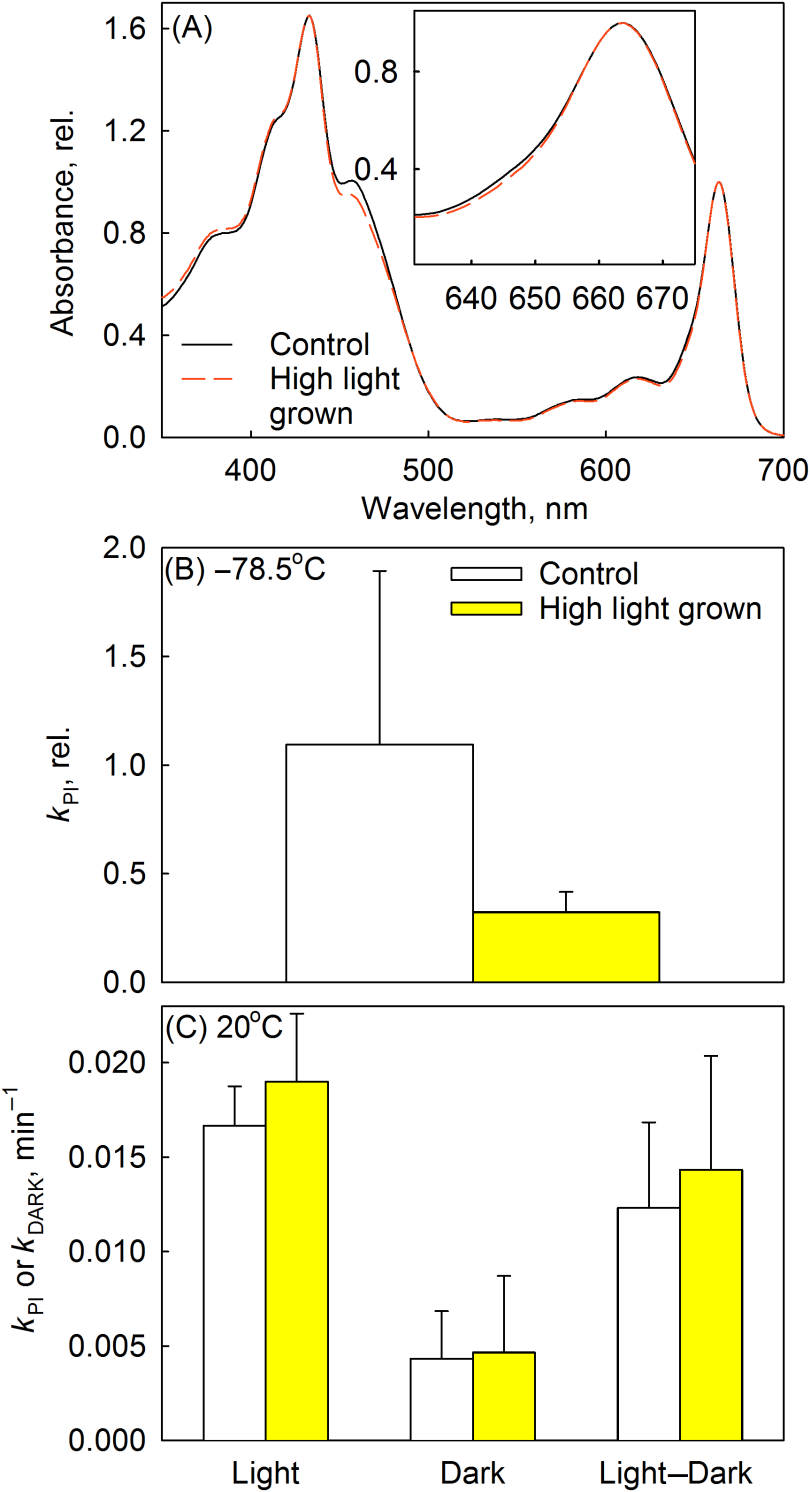
Effect of antenna size on photoinhibition. (A) Absorption spectra, normalized to 665 nm, of thylakoid (in acetone; dashed lines) extracts. Thylakoids were isolated from leaves of *Arabidopsis* grown under control (continuous black line) or high light conditions (red dashed line). The inset shows the 630–680 nm range in more detail. (B) Thylakoids from control (open bars) or high‐light *Arabidopsis* (yellow bars) were illuminated (photosynthetic photon flux density [PPFD] 2000 μmol m^−2^ s^−1^) at −78.5°C for 60 min. Rate constants of photoinhibition (*k*_PI_) were calculated assuming that a decline in the photosystem II (PSII) oxygen evolution rate (H_2_O to dimethylbenzoquinone) followed the kinetics shown in Figure 2B. The rate constants are normalized to that obtained by modeling the result from Figure 2B. For original PSII activities, see Figure S5. (C) Thylakoids from control or high‐light *Arabidopsis* were illuminated (PPFD 2000 μmol m^−2^ s^−1^; Light) or incubated in the dark (Dark) at 20°C for 45 min. Rate constants were calculated assuming that a decline in the PSII oxygen evolution rate followed, both in the light and in darkness, first‐order reaction kinetics; rate constants of photoinhibition (*k*_PI_; Light–Dark) were obtained by subtracting the rate of dark‐inactivation (*k*_DARK_) from the rate of light‐induced decline. Each bar represents an average of at least three independent repetitions and the error bars show SD.

Finally, to test the effect of antenna size on low‐temperature photoinhibition, thylakoids were isolated from *Arabidopsis* grown at 20°C either under control (PPFD 150–200 μmol m^−2^ s^−1^) or under high light (PPFD 1000 μmol m^−2^ s^−1^) conditions. The chlorophyll *a*/*b* ratio was higher in thylakoids isolated from plants grown in high light (3.89 ± 0.13) than in the control thylakoids (3.39 ± 0.54; *p* = 0.017), consistent with earlier data showing that a decrease in PSII antenna size is a common response to high light in *Arabidopsis* (Ballottari et al., 2007). On the other hand, the carotenoid‐to‐chlorophyll ratio did not increase in response to high light (Figure 6A).

When *Arabidopsis* thylakoids were illuminated at −78.5°C, photoinhibition proceeded faster in control thylakoids than in the high‐light thylakoids, though the difference was not statistically significant (Figure 6B). On the contrary, when the illumination was conducted at 20°C, thylakoids isolated from high light‐grown *Arabidopsis* were not more resistant against photoinhibition than control thylakoids (Figure 6C).

The finding that thylakoids of high light‐grown plants were not more resistant than control thylakoids against photoinhibition at 20°C (Figure 6C), in turn, agrees with earlier data showing that antenna size does not affect photoinhibition in vitro at 20°C (Tyystjärvi et al., 1994).

## 4 DISCUSSION

Photoinhibition of PSII was found to proceed even at −78.5°C. When induced by illumination with visible light, photoinhibition at −78.5°C depended on oxygen (Figure 2A), suggesting that the damaging agent is a reactive oxygen species. Chemical reactions are required for the formation of oxygen radicals, which strongly suggests that singlet oxygen, rather than an oxygen radical, is the main reactive oxygen species formed at −78.5°C. Photoinhibition at −78.5°C, unlike photoinhibition at above‐zero temperatures (for a review, see Tyystjärvi, 2013), did not proceed with first‐order reaction kinetics but slowed down with time (Figure 2B), suggesting that conversion to singlet oxygen during illumination depletes the vicinity of PSII of oxygen in the frozen sample, where diffusion of oxygen is limited (Hemmingsen, 1959). The oxygen‐dependent kinetics of photoinhibition at −78.5°C required a new type of photoinhibition model (Figure S1) that was able to reproduce the observed kinetics (Figure 2B). Due to the large number of parameters that could not be measured, the model was used only to extract relative values of the rate constant of photoinhibition for comparison of different samples. When fixing all but one rate constant of the model for the analysis of fixed‐time assays, we formally assumed that the initial rate of photoinhibition is directly proportional to the initial rate of singlet oxygen production, but the intrinsic susceptibility of PSII to singlet oxygen, the rate constant of conversion of singlet oxygen to O_2_ and loss of singlet oxygen via unspecified oxidations remain constant. However, the model cannot distinguish between the different factors causing differences in the loss of PSII activity during illumination at −78.5°C, and therefore the fitted values of the intrinsic rate constant of the model only represent relative values that allow the rate constant of photoinhibition to be compared between relatively similar samples, like thylakoid samples from one species.

*Bergenia* thylakoids, isolated from winter leaves, produced less singlet oxygen at 20°C than thylakoids isolated from summer leaves and were resistant to photoinhibition of PSII both at −78.5°C and at 20°C (Figure 4). These results lend support to earlier data suggesting that singlet oxygen is involved in photoinhibition (e.g., Davis et al., 2016; Fufezan et al., 2002, 2007; Rehman et al., 2013; Treves et al., 2016). However, photoinhibition at 20°C proceeded also in the absence of oxygen (Figure 2), indicating that oxidation by singlet oxygen is not the only mechanism of photoinhibition at 20°C. Similar findings have earlier led to the suggestion that photoinhibition at physiological temperatures proceeds via several parallel mechanisms (Mattila et al., 2022; Oguchi et al., 2009; Tyystjärvi, 2013). The observation also shows that different photoinhibition mechanisms dominate at 20°C than at −78.5°C. The conclusion is further supported by the observations that thylakoids isolated from high light‐grown *Arabidopsis* were tolerant against photoinhibition at −78.5°C but not at 20°C (Figure 6), and that the *Synechocystis* ΔrpoZ strain was not more tolerant against photoinhibition at −78.5°C than the control strain (Figure 5), even though the ΔrpoZ strain produces little singlet oxygen and is tolerant against photoinhibition at 32°C (Hakkila et al., 2013). Furthermore, this result indicates that the mechanisms protecting the ΔrpoZ strain against photoinhibition at 32°C do not function at a very low temperature.

The dependence of photoinhibition on oxygen at −78.5°C (Figure 2) suggests the involvement of singlet oxygen. Charge recombination reactions would be an obvious candidate for the process producing triplet chlorophyll (and consequently singlet oxygen) but, at −78.5°C, recombination of the primary charge pair, P_680_^+^Pheo^−^, right after its formation by charge separation, is the only PSII recombination reaction that can lead to triplet chlorophyll production and has a significant rate (see Rappaport & Lavergne, 2009; Zabelin et al., 2016). In addition, chlorophyll triplets produced by intersystem crossing in the antennae might be important for singlet oxygen production and photoinhibition at −78.5°C. At cryogenic temperatures, chlorophyll triplets distinct from the charge recombination triplet (^3^P_680_) are detected (Santabarbara et al., 2001, 2003, 2007) and it has been suggested that uncoupled chlorophylls in antennae cause PSII photoinhibition (Santabarbara et al., 2001). At room temperature, antenna chlorophyll triplets are efficiently quenched by carotenoids, which diminish singlet oxygen production (Mozzo et al., 2008). At lower temperatures, however, a sub‐population of antenna chlorophylls has been observed to become energetically detached and not quenched at all (Vinklárek et al., 2018). The finding that thylakoids isolated from high light‐grown *Arabidopsis*, having smaller antennae than control plants, were more resistant against photoinhibition at −78.5°C than control thylakoids (Figure 6B), does not distinguish the reaction center and the antennae as singlet oxygen producers, as both would diminish with diminishing antenna size. However, the finding that photoinhibition at −78.5°C proceeded more slowly in the presence of a strong external magnetic field (Figure 3C) suggests that singlet oxygen produced via recombination of the primary charge pair is the main contributor to the observed photoinhibition at −78.5°C. The lack of a magnetic field effect on singlet oxygen production at 25°C, on the other hand, does not exclude PSII recombination reactions as the source of the triplet at 25°C, as recombinations involving Q_A_^−^ (possible at 25°C though not at −78.5°C) start with noncorrelated spins, and their triplet yield would therefore be independent of a magnetic field. In particular, the miss‐associated recombination of P_680_^+^Q_A_^−^ has been shown to be important at above‐zero temperatures (Mattila et al., 2022).

Whether singlet oxygen originating in antennae contributed to photoinhibition at −78.5°C is difficult to judge from the present data. The observation that increased carotenoids in the *Synechocystis* ΔrpoZ strain did not protect against photoinhibition at −78.5°C (Figure 5) may suggest that diffusion of singlet oxygen out of the site of its production may be limited in a frozen state, and therefore the carotenoids may have less chance to quench the singlet oxygen in *Synechocystis* that is not adapted to survival at very low temperatures. Thus, it is possible that antenna‐originating singlet oxygen never reaches the PSII reaction center at −78.5°C. Carotenoids are obviously important for evergreen species, as it has been reported that amounts of carotenoids, such as lutein and xanthophylls, increase during winter in evergreen plants like *Rhododendron* (B. Liu et al., 2019). Also in *Bergenia* leaves, the carotenoid‐to‐chlorophyll ratio increased during winter (Figure 4). In general, many mechanisms protecting against reactive oxygen species are enhanced during winter in evergreen species (X. Wang et al., 2009).

The behavior of PSII antenna proteins during winter in evergreen species is variable. Míguez et al. (2017) showed that amounts of the PSII antenna protein lhcb2 were lower during winter in some evergreen species (*Hieracium pilosella* and *Syntrichia muralis*), whereas the amounts stayed constant thorough the year in other species (*Cytisus cantabricus*). In *Haberlea rhodopensis*, different LHCII proteins had different fates during winter but, in general, the LHCII/PSII ratio increased (Mihailova et al., 2020). In evergreen spruce and pine, the amount of LHCII seems to remain constant during winter, or at least the decrease is smaller than that of the D1 reaction center protein (Ebbert et al., 2005; Ensminger et al., 2004). These data indicate that a small PSII antenna is not a universal response to low temperature. Also, *Bergenia* rather showed higher chlorophyll *a‐*to‐*b* ratios during summer (3.12 ± 0.06 in leaves and 3.02 ± 0.30 in thylakoids) than during winter (2.74 ± 0.05 in leaves and 2.87 ± 0.05 in thylakoids). It should be noted that, with the present methodology, it is not possible to detect changes in the level of individual carotenoid species or subtle changes in antenna structures. It can be speculated, however, that instead of antenna degradation for winter, the LHCII of *Bergenia* and other evergreen species may become quenched, which would prevent the antenna from producing singlet oxygen. Quenching of excitation energy has been shown to be a part of winter acclimation in many evergreen species (Adams & Demmig‐Adams, 1994; Bag et al., 2020; Demmig‐Adams et al., 2015; Grebe et al., 2020; Verhoeven et al., 1996). In spruce, direct energy transfer (spillover) from PSII to PSI, as well as functional detachment of some LHCII, were proposed to explain the sustained NPQ (Bag et al., 2020).

Besides visible light, UV radiation also induced photoinhibition at −78.5°C, even in the absence of oxygen (Figure 2). At temperatures above zero, PSII damage by UV radiation occurs via the release of a Mn ion from the OEC due to UV absorption of the Mn ions (Hakala et al., 2005; Renger et al., 1989; Vass et al., 1996). Photoinhibition induced by UV radiation has a weak positive temperature dependence between 4 and 22°C (Lazarova et al., 2014; Mattila et al., 2022), with an activation energy of 0.09 eV (Mattila et al., 2022), and direct application of the Arrhenius equation predicts that its rate constant at −78.5°C is 16.5% of that measured at 20°C, if other conditions are equal. However, the large optical differences between liquid water and ice (Figure 1) do not allow a direct comparison of the rate constants.

## AUTHOR CONTRIBUTIONS

The experiments were designed by Esa Tyystjärvi and Heta Mattila. Heta Mattila conducted the research. Esa Tyystjärvi build the models. Heta Mattila wrote the original draft of the manuscript. Esa Tyystjärvi contributed to the final version.

## Supporting information


**Figure S1.** Model of the kinetics of photoinhibition at −78.5°C.
**Figure S2**. Oxygen evolution activities of pumpkin thylakoids before and after an illumination or a dark treatment either at −78.5°C or 20°C, in aerobic or anaerobic conditions.
**Figure S3**. Oxygen evolution activities of pumpkin thylakoids before and after an illumination or a dark treatment at −78.5°C in aerobic conditions, in the presence or absence of a strong external magnetic field.
**Figure S4**. Oxygen evolution activities of summer and winter thylakoids of *Bergenia* before and after an illumination or a dark treatment at −78.5°C, in aerobic or anaerobic conditions.
**Figure S5**. Oxygen evolution activities of thylakoids of control and high light grown *Arabidopsis* before and after an illumination or a dark treatment at −78.5°C in aerobic conditions.
**Appendix S1**. Results of a linear statistical model for the decrease of the D1 protein in pumpkin thylakoids illuminated in dry ice (−78.5°C) and then incubated at 20°C in the presence of GTP.

## Data Availability

Original data are available at “Photoinhibition at 195 K”, Mendeley Data, V1, doi: 10.17632/74jcfswp8d.1.
