# Supplementary material for: Light‐induced damage to photosystem II at a very low temperature (195 K) depends on singlet oxygen
Source: Physiol Plant. 2022 Nov 28;174(6):e13824. doi: 10.1111/ppl.13824 (PMC10099935; doi:10.1111/ppl.13824)
Supplement: Supplementary file 1 — Figure S1. Model of the kinetics of photoinhibition at −78.5°C. Figure S2. Oxygen evolution activities of pumpkin thylakoids before and after an illumination or a dark treatment either at −78.5°C or 20°C, in aerobic or anaerobic conditions. Figure S3. Oxygen evolution activities of pumpkin thylakoids before and after an illumination or a dark treatment at −78.5°C in aerobic conditions, in the presence or absence of a strong external magnetic field. Figure S4. Oxygen evolution activities of summer and winter thylakoids of Bergenia before and after an illumination or a dark treatment at −78.5°C, in aerobic or anaerobic conditions. Figure S5. Oxygen evolution activities of thylakoids of control and high light grown Arabidopsis before and after an illumination or a dark treatment at −78.5°C in aerobic conditions. Appendix S1. Results of a linear statistical model for the decrease of the D1 protein in pumpkin thylakoids illuminated in dry ice (−78.5°C) and then incubated at 20°C in the presence of GTP. [file PPL-174-0-s001.docx]

**At a very low temperature (195 K) light-induced damage to Photosystem II depends on singlet oxygen**

Heta Mattila, Esa Tyystjärvi*

University of Turku, Department of Life Technologies / Molecular Plant Biology, Itäinen Pitkäkatu 4 C 6^th^ floor, 20520 Turku, Finland

*Corresponding author, email: esatyy@utu.fi

**Supporting Information**


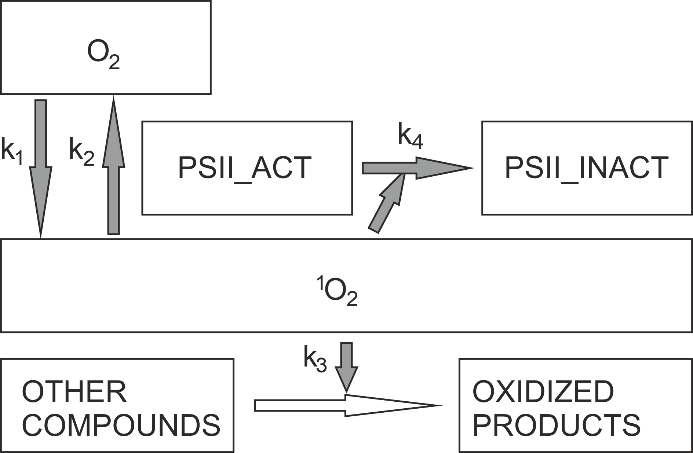


Figure S1. Kinetic model of photoinhibition at -78.5°C. The rate constants k_1_ to k_4_ are associated with the grey arrows; k_1_, k_2_ and k_3_ are first-order constants and k_4_ is a second-order rate constant. The initial pool sizes of O_2_ and PSII were assumed to be 0.28 µmol and 0.28 nmol, respectively, and the net rate constants of conversion of O_2_ to singlet oxygen (^1^O_2_) (k_1_) and the reverse conversion (k_2_), loss of ^1^O_2_ via unspecified reactions (k_3_) and photoinhibition of PSII (k_4_) were originally let to run free. For the estimation of the rate constant of photoinhibition from fixed-time assays, rate constants k_2_, k_3_ and k_4_ were fixed to the values obtained from the determination of the kinetics (0.0024 s^-1^, 3.45 s^-1^ and 7284 s^-1^ M^-1^).

**
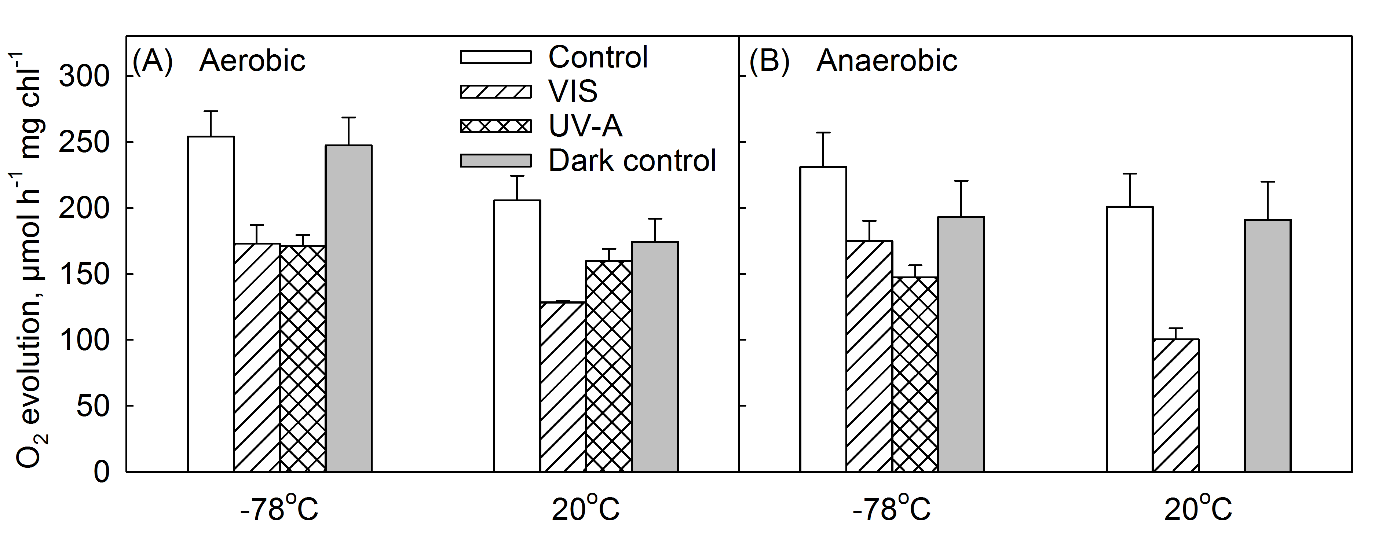
**

Figure S2. PSII oxygen evolution (H_2_O to DMBQ) activity in isolated thylakoid membranes of pumpkin. Thylakoid membranes were illuminated with white light (VIS; PPFD 1150 µmol m^-2^ s^-1^) for 40 min (open hatched bars), with UV-A radiation (PFD 300 µmol m^-2^ s^-1^) for 10 min (open cross-hatched bars) or frozen/melted in the dark (Dark control; grey bars), either at -78.5°C or at 20°C, as indicated, in aerobic (A) or anaerobic (B) conditions. The data show averages from at least three independent repetitions and the error bars show SD.


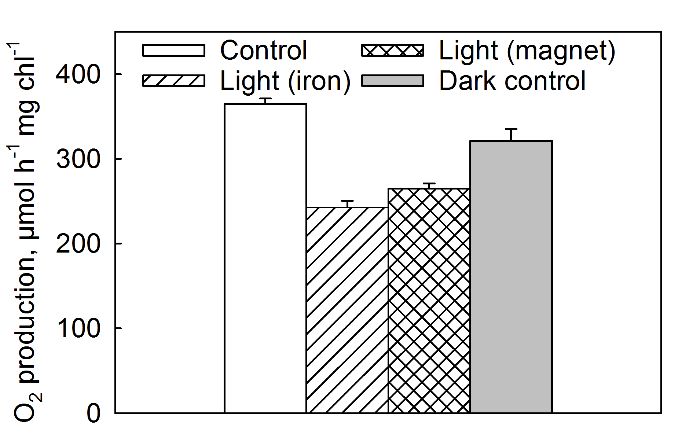


Figure S3. PSII oxygen evolution (H_2_O to DMBQ) activity in isolated thylakoid membranes of pumpkin. Thylakoid membranes were illuminated with white light (PPFD 1150 µmol m^-2^ s^-1^) for 40 min (Light), either between iron blocks (hatched bars) or magnets (in 170 mT magnetic field; cross hatched bars), or frozen/melted in the dark (Dark control; grey bars) at -78.5°C in aerobic conditions. The data show averages from at least three independent repetitions and the error bars show SD.


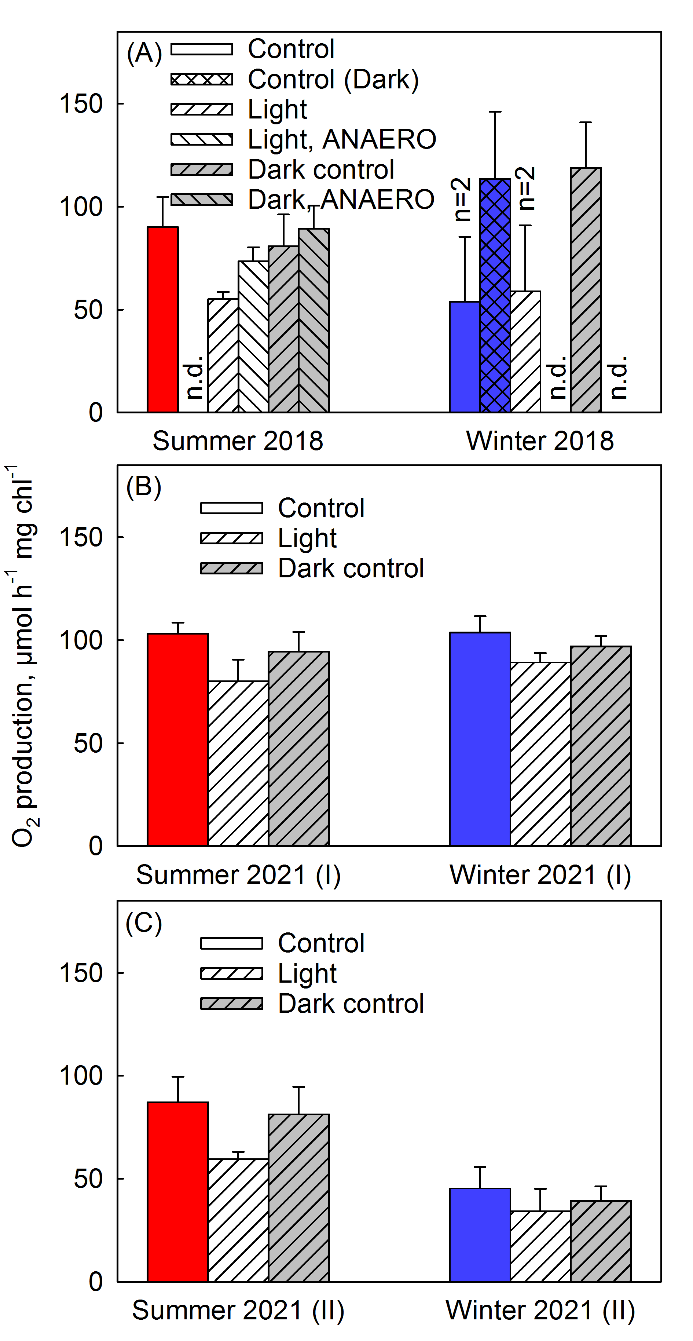


Figure S4. PSII oxygen evolution (H_2_O to DMBQ) activity in thylakoid membranes of *Bergenia*, isolated from leaves collected either during winter or summer, as indicated, during 2018 (A) and 2021 (B–C). The Roman numbers I and II after 2021 refer to spring and autumn, respectively. Thylakoid membranes were illuminated with white light (PPFD 2000 µmol m^-2^ s^-1^) for 120 min (Light; open hatched bars) or frozen/melted in the dark (Dark control; grey hatched bars) at -78.5°C in aerobic or anaerobic (ANAERO) conditions. In the case of 2018 winter thylakoids, control measurements were done separately for dark and light treatments. n.d. = not determined. The data show averages from at least three independent repetitions, unless otherwise specified, and the error bars show SD.


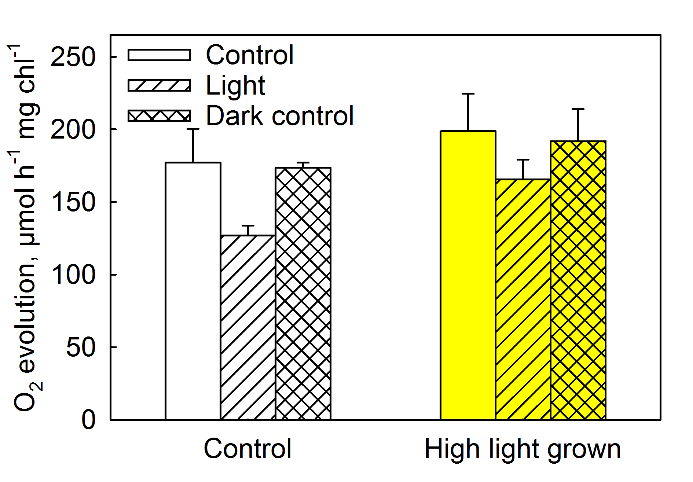


Figure S5. PSII oxygen evolution (H_2_O to DMBQ) activity in isolated thylakoid membranes of *Arabidopsis* grown under control (open bars) or high light conditions (yellow bars). Thylakoid membranes were illuminated with white light (PPFD 2000 µmol m^-2^ s^-1^) for 60 min (Light; hatched bars) or frozen/melted in the dark (Dark control; cross-hatched bars) at -78.5°C in aerobic conditions. The data show averages from at least three independent repetitions and the error bars show SD.

**Appendix 1: Results of a linear statistical model for the decrease of the D1 protein in thylakoids illuminated in dry ice and then incubated at 20°C in the presence of GTP**

**R function call**

lm(formula = D1_amount ~ Illumination_time * Dark_time, data = D1data)

**Residuals**

| **Min** | **1Q** | **Median** | **3Q** | **Max** |
| --- | --- | --- | --- | --- |
| -16.9657 | -4.9294 | 0.0399 | 2.5819 | 15.1244 |

**Coefficients**

|  | **Estimate** | **Std. error** | **t value** | **Pr (>\|t\|)** | **Significance** |
| --- | --- | --- | --- | --- | --- |
| **(Intercept)** | 99.960095 | 2.429475 | 41.145 | <2E-16 | *** |
| **Illumination_time** | -2.551661 | 1.396366 | -1.827 | 0.0791 | . |
| **Dark_time** | 0.003041 | 0.717646 | 0.004 | 0.9967 |  |
| **Illumination_time*Dark_time** | -1.471732 | 0.711018 | -2.070 | 0.0485 | * |

Residual standard error: 7.14 on 26 degrees of freedom.

Multiple r^2^: 0.5328, Adjusted r^2^: 0.4789.

F-statistic: 9.883 on 3 and 26 DF, p-value: 0.0001591.


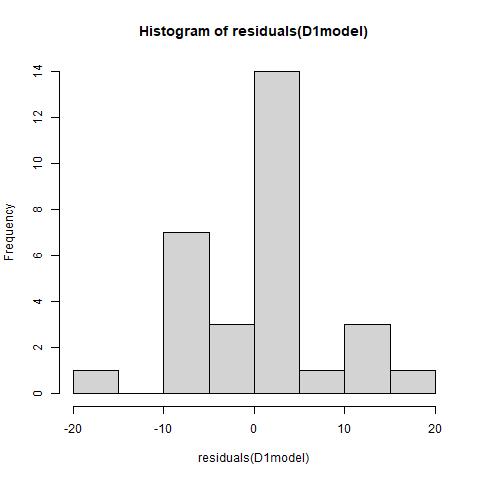


Fig. S6. Residuals of the linear model for the amount of the D1 protein.


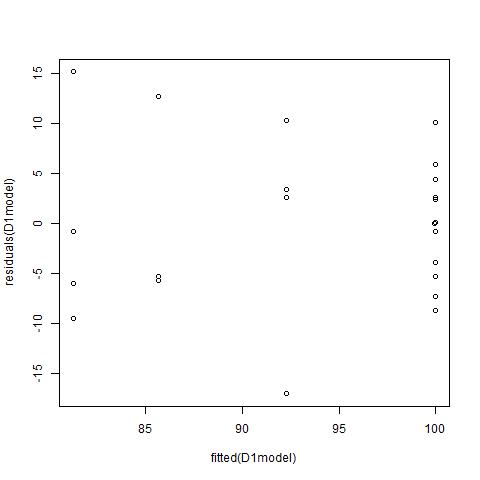


Fig. S7. Residuals of the linear model for the amount of the D1 protein as a function of the fitted values of the model.


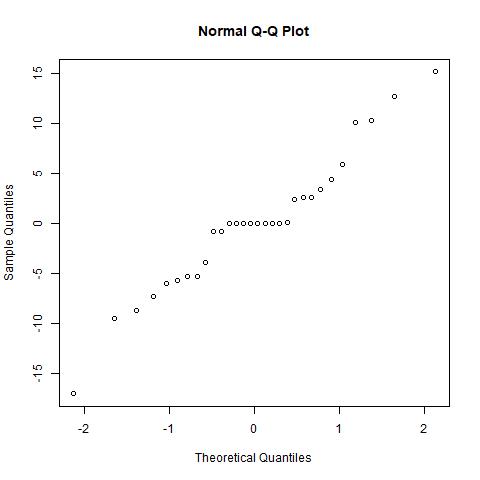


Fig. S8. Comparison of the sample quantiles of the model for the amount of the D1 protein with the theoretical quantiles.
